# Partial splenic embolization combined with endoscopic therapies and NSBB decreases the variceal rebleeding rate in cirrhosis patients with hypersplenism: a multicenter randomized controlled trial

**DOI:** 10.1007/s12072-021-10155-0

**Published:** 2021-02-27

**Authors:** Xin Sun, Anzhong Zhang, Tao Zhou, Minghui Wang, Yong Chen, Ting Zhou, Xiaoning Chen, Aiyuan Xiu, Zhi Peng, Baoquan Cheng, Xiaofeng Liu, Yanjing Gao

**Affiliations:** 1 Department of Gastroenterology, Qilu Hospital of Shandong University, Wenhua Xi Road, 107, Jinan City, 250012 Shandong Province People’s Republic of China; 2 Department of Gastroenterology, Shandong Provincial Hospital Affiliated to Shandong University, Jinan City, 250021 Shandong Province China; 3 Department of Gastroenterology, The 960th Hospital of the PLA Joint Logistics Support Force, Shifan Road, 25, Jinan City, 250031 Shandong Province People’s Republic of China

**Keywords:** Liver cirrhosis, Partial splenic embolization, Hypersplenism, Thrombocytopenia, Portal hypertension, Gastroesophageal varices, Secondary prophylaxis, Endoscopic variceal ligation, Non-selective beta-blocker, Cyanoacrylate injection

## Abstract

**Background:**

Global research on endoscopic therapies in combination with partial splenic embolization (PSE) for variceal hemorrhage (VH) is limited. Therefore, we aimed to evaluate the efficacy and safety of endoscopy plus PSE (EP) treatment in comparison to endoscopic (E) treatment for the secondary prophylaxis of VH in cirrhosis patients with hypersplenism.

**Methods:**

Cirrhosis patients with hypersplenism (platelet count < 100, 000/µL) and those who had recovered from an episode of VH were enrolled in a multicenter randomized controlled trial. The participants were randomly assigned into EP and E groups in a 1:1 ratio. The primary endpoint was variceal rebleeding, and the secondary endpoints were severe variceal recurrence and mortality during the 2-year follow-up. Hematological indices, serum biochemical parameters, and the Child–Pugh score were measured at each time point.

**Results:**

From June 2016 to December 2019, 108 patients were enrolled in the study, among which 102 patients completed the protocol (51 in EP and 51 in E group). The rebleeding rate of the varices was significantly reduced in the EP group compared to that in the E group during the 2 years (16% vs. 31%, *p* < 0.001). The EP group showed a significantly lower variceal recurrence rate than the E group (22% vs. 67%, *p* < 0.001). The COX proportional hazard models revealed that grouping was an independent predictor for variceal rebleeding (*H* = 0.122, 95% CI 0.055–0.270, *p* < 0.001) and variceal recurrence (hazard ratio, *H* = 0.160, 95% CI 0.077–0.332, *p* < 0.001). The peripheral blood cell count, Child–Pugh class/score, albumin concentration, and coagulation function in the EP group improved significantly compared to the values observed in the E group at any time point (*p* < 0.05).

**Conclusions:**

The EP treatment was more effective in preventing variceal rebleeding and variceal recurrence than the conventional E treatment during the secondary prophylaxis of VH in cirrhosis patients with hypersplenism. Furthermore, the EP treatment could significantly increase the peripheral blood cell count and albumin concentration and also improved the coagulation function and the Child–Pugh score.

**Clinical trials registration:**

Trial registration number ClincialTrials.gov: NCT02778425. The URL of the clinical trial: https://clinicaltrials.gov/

**Supplementary Information:**

The online version contains supplementary material available at 10.1007/s12072-021-10155-0.

## Introduction

Varices and variceal hemorrhage (VH) are the major complications of portal hypertension that cause high mortality in cirrhosis patients. Nearly half of the newly diagnosed patients with compensated cirrhosis develop gastroesophageal varices (GEVs) [1]. The annual risk of the first VH is approximately 5–15% and entails 6-week mortality of 10–20% [2]. The 1-year risk of rebleeding after the first episode of bleeding is 60%, and the mortality rate is up to 33% [3, 4]. Hence, secondary prophylaxis is necessary to prevent rebleeding in cirrhosis patients who had recovered from an episode of acute VH.

According to the Baveno VI recommendations, non-selective beta-blocker (NSBB) combined with endoscopic variceal ligation (EVL) is recommended for the secondary prophylaxis of esophageal variceal hemorrhage. Cyanoacrylate injection in the patients with large GEV type 2 or isolated gastric varices (GV) type 1 is promising for the prevention of first variceal bleeding [5]. A retrospective cohort study demonstrated the efficacy of continued EVL plus cyanoacrylate injection for the secondary prophylaxis of variceal bleeding in patients with concomitant esophageal varices (EV) and GV [6]. However, the application of cyanoacrylate in these patients should be investigated further with the evaluation of the risk/benefit ratio.

The incidence of hypersplenism in cirrhosis patients with portal hypertension is 11%–55% [7]. Hypersplenism is a major contributing factor in the development of thrombocytopenia, anemia, and leukopenia in such patients. Hypersplenism often develops in parallel with splenomegaly. Clinically, splenomegaly in cirrhosis patients is associated with a poor prognosis along with the maintenance of portal hypertension due to increased splenic blood flow. Moreover, thrombocytopenia and splenomegaly are independent predictors of bleeding from large varices [8]. Partial splenic embolization (PSE) is effective in improving the hematologic parameters, reducing the episodes of variceal bleeding, maintaining long-term eradication of the varices, and enhancing hepatic synthesis [9–11]. Unfortunately, there is no universally accepted therapy for GEVs in cirrhosis patients with hypersplenism, especially those with thrombocytopenia.

However, on the theoretical basis mentioned above, we consider that a combined therapy involving endoscopic therapies and PSE for GEVs in cirrhosis patients with hypersplenism would be sufficient. Therefore, we conducted a prospective study to assess the efficacy of EVL and cyanoacrylate injection combined PSE (EP treatment) vs. EVL and cyanoacrylate injection (E treatment) alone for the secondary prophylaxis of VH in cirrhosis patients with hypersplenism.

## Methods

### Study design and patients

The present study was a prospective, multicenter, double-blind, randomized controlled trial. The study was carried out at the Gastroenterology Department of three tertiary referral hospitals (Qilu Hospital of Shandong University, the 960th Hospital of the PLA Joint Logistics Support Force, and Shandong Provincial Hospital Affiliated to Shandong University) that treat most of the patients with liver cirrhosis in China. The study was approved by the Ethics Committee of Shandong University. Each institution approved the protocol, and the data of all patients were recorded in a standard form. All patients were informed of the aims of the study and signed the informed consent before participation. This study was registered at ClinicalTrials.gov with the identifier number: NCT02778425.

The enrollment criteria were: (i) patients aged between 18 and 75 years; (ii) patients who had recovered from an episode of VH or patients who had survived from acute VH and there was no bleeding for consecutive 5 days; (iii) patients with a diagnosis of liver cirrhosis and portal hypertension on clinical examination, laboratory test, and imaging or histological examination; and (iv) patients with hypersplenism and thrombocytopenia (platelets < 100,000/µL).

The exclusion criteria were: (i) previous therapy (splenectomy, PSE, EVL, tissue adhesive injection, or usage of NSBB) to prevent rebleeding; (ii) bleeding from isolated gastric or ectopic varices; (iii) hepatocellular carcinoma or other malignant tumors; (iv) contraindications for the use of NSBBs, hepatic failure, and Child–Pugh class C with large amount ascites, or grade 3–5 hepatic encephalopathy, or prothrombin activity ≤ 40%; (v) hepatic failure; (vi) contraindications for PSE; (vii) pregnancy and lactation; and (viii) inability to sign the informed consent.

### Randomization and masking

The patients qualifying the inclusion criteria for this study were randomly assigned into EP and E groups in a 1:1 ratio. Randomization was carried out according to a computer-generated randomization sequence. Patient allocation and assignment were performed using a sealed opaque envelope. The endoscopist, interventional radiologist, and trial statistician were blinded to the treatment assignments.

### Endoscopic treatment protocols

On the premise of hemodynamic stability, the patients underwent endoscopic examination and endoscopic treatment after receiving intravenous anesthesia under the supervision of an anesthesiologist. All the diagnostic and therapeutic endoscopies were performed using PENTAX Gastroscopy (EG29-i10, PENTAX) by a technically proficient gastrointestinal endoscopist and the support staff. During the first session, cyanoacrylate (Histoacryl glue, Braun, Melsungen, Germany, or Beijing Component Medical Devices Co. Ltd.) was injected using a 23-gauge disposable injection needle (NM-200L-0521, Olympus, Tokyo, Japan) into the GV following the standardized sandwich method (normal saline + cyanoacrylate + normal saline). Each shot contained no more than 2.0 mL cyanoacrylate plus an equal volume of normal saline. In each session, no more than six injections were administered. Furthermore, EVL was carried out with a 6 Shooter Saeed Multi-Band Ligator (Cook Endoscopy, Inc., Winston-Salem, NC, USA) at 1 cm above the Z-line in a spirally ascending fashion.

After the initial session, EVL was performed at regular intervals of about 4 weeks. If necessary, additional cyanoacrylate was injected into distinct large GV until the varices were eradicated.

### PSE protocols

PSE was performed 1 week before the endoscopic treatment in the EP group. All patients underwent enhanced computed tomography (CT) scanning to observe the basal status of the spleen. Initially, splenic arteriography was performed on a digital subtraction angiography (DSA) system via the femoral artery access. Using the Seldinger technique to apply a 5 French guiding catheter (Terumo, Tokyo, Japan) into the femoral artery, the contrast agent was administered to demonstrate the splenic artery and the collateral circulation routes. A microcatheter was located distally in the splenic hilus by superselective catheterization. Progressive embolization was performed by repeated injections of a gelatin sponge cut into 1–2-mm cubes under the DSA control until a 60–80% reduction in the splenic blood flow was achieved.

Further observation and management for all the patients were performed in the hospital. Broad-spectrum antibiotic prophylaxis was administered in the perioperative period. A post-interventional enhanced CT scan was performed to confirm the embolization area 2–3 weeks after the PSE.

### Additional treatment

A standard dose of NSBB (propranolol) was applied to patients according to the Baveno VI recommendations if there were no contraindications. The dosage of propranolol was titrated to either reduce the resting heart rate by 25% or achieve a heart rate of up to 55 beats /min, or a maximum tolerated dose was used. All the post-hepatitis B cirrhotic participants received anti-viral therapy.

### Outcomes and definitions

The primary endpoint of the study was variceal rebleeding during the 2-year follow-up. The secondary endpoints were severe variceal recurrence and mortality. Hematological indices, serum biochemical parameters, and the Child–Pugh score were measured at each time point.

Eradication of the varices was defined as no visible varices in endoscopy or the presence of only fibrosed varices not requiring ligation. Variceal bleeding was defined as active hematemesis and/or melena, presence of active esophageal or esophagogastric variceal bleeding in endoscopy, red signs of varix, or with plug overlying varix. Recurrence of varices referred to the detection of new varices after variceal eradication had been previously achieved.

### Follow-up

After variceal eradication was achieved, a routine follow-up was performed for each patient at the time points of 3 months, 6 months, 1 year, and 2 years. The follow-up visit included clinical assessment, endoscopic follow-up, evaluation of hematological parameters, serum biochemical analysis, and Child–Pugh score. Follow-up endoscopy was performed every 3 months, and if there was no recurrence, after every 6 months. Once the patients suffered from variceal bleeding or generated new varices with forms F2/F3 [12] as observed in endoscopy, they were dropped out of the group.

### Statistical analysis

Statistical analysis was performed using the Statistical Program for Social Science (SPSS) version 11.0 for Windows (SPSS, Chicago, IL). The sample size was estimated based on the varices rebleeding rate in the secondary prophylaxis. Assuming the rebleeding rate to be 37% in the E group and 15% in the EP group, and 5% α and 10% β error, respectively, approximately 110 patients were included considering a 20% dropout rate.

The continuous variables were expressed as mean ± standard deviation (SD) or (interquartile range)/M (IQR) and analyzed by two-sample Student’s *t* tests or Mann–Whitney *U* test for comparison. The categorical variables were expressed as frequency and percentage and were analyzed using the Chi-squared test.

The rebleeding and recurrence rates of the varices were analyzed by the Kaplan–Meier survival curve. The log-rank test was used for a comparison between the two groups. The Cox univariate analysis was performed to assess the candidate variables significantly (*p* < 0.20). Next, the candidate variables, along with the other variables that were thought to be necessary for contributions, were included in the Cox multivariate analysis to determine the independent factors predicting variceal rebleeding and variceal recurrence. A *p*-value of less than 0.05 was considered statistically significant.

## Results

### Characteristics and follow-up of the patients

**Fig. 1:**
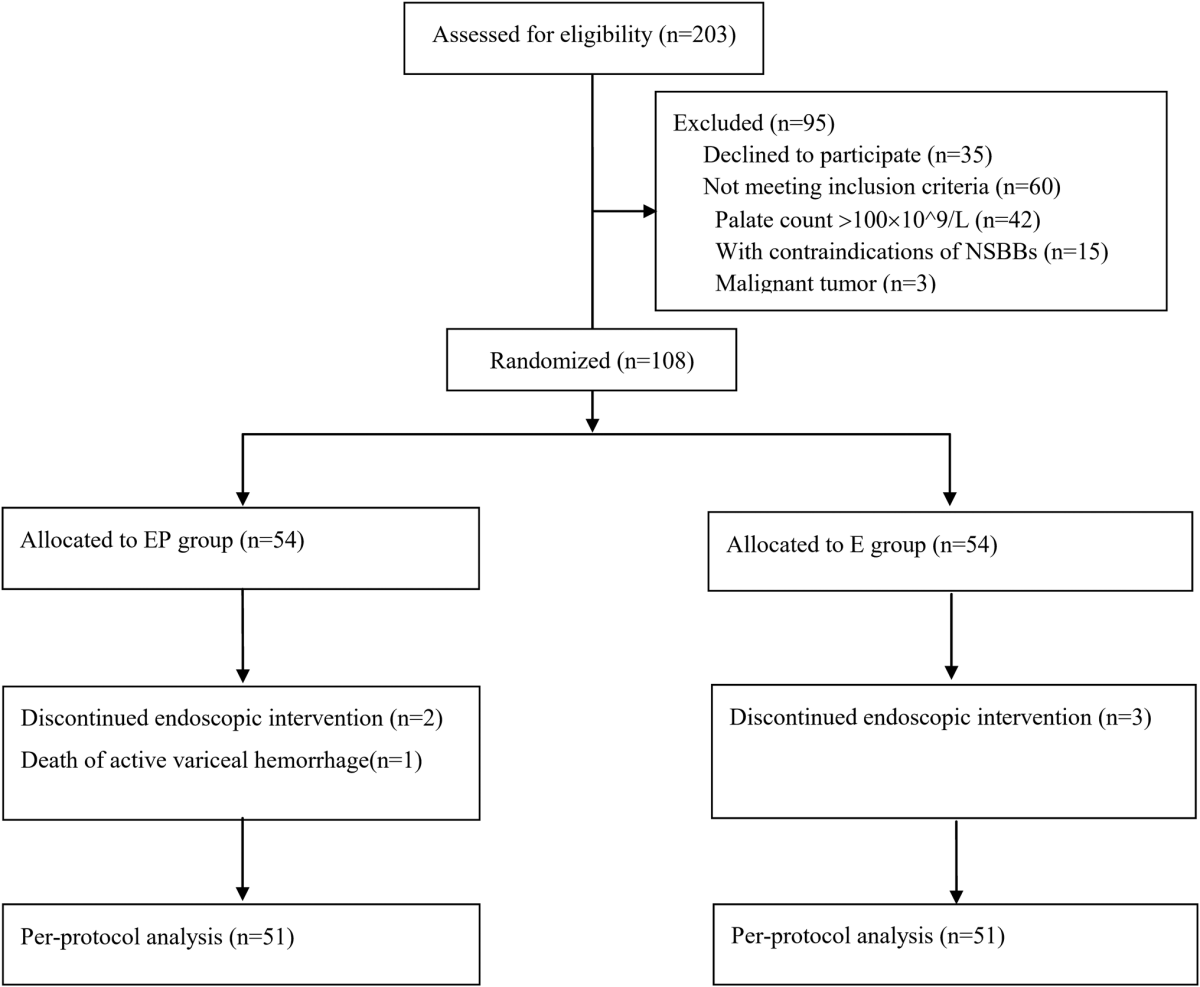
Flow diagram of the study. *PSE* Partial splenic embolization

From June 2016 to December 2019, 108 patients were enrolled in the randomized trial and divided into two groups in a 1:1 ratio. Two patients from the EP group and three from the E group were discontinued from the endoscopic treatment because of poor compliance. One patient from the EP group died due to acute variceal bleeding induced by discontinued endoscopic treatment and hard food consumption 2 months after the PSE. Finally, 51 patients from the EP group and 51 patients from the E group completed the per-protocol analysis (Fig. 1).

The baseline characteristics shown in Table 1 were well-matched (*p* > 0.05) in the two groups. Two patients from the EP group and three patients from the E group were discontinued from the NSBB treatment due to poor compliance (*p* > 0.05).

### Variceal rebleeding

**Fig.2:**
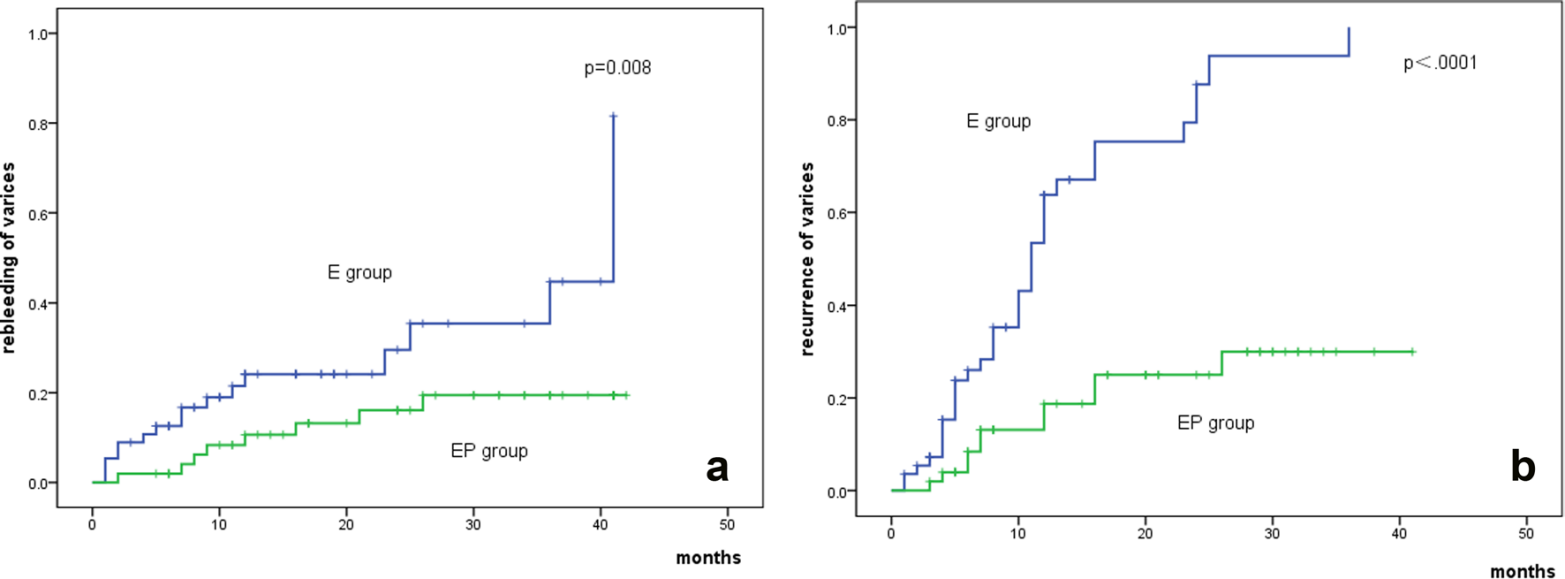
Kaplan–Meier curves of variceal rebleeding (**a**), variceal recurrence (**b**). The log-rank *p* values of which were 0.000 and 0.008, respectively; *PSE* partial splenic embolization

After the initial endoscopic or EP treatment, variceal rebleeding occurred in a total of 24 patients, including eight patients in the EP group and 16 patients in the E group (16% vs. 31%, log-rank *p* = 0.008). The Kaplan–Meier curve for rebleeding is presented in Fig. 2a. The median variceal rebleeding period was 41 months in the E group. According to the COX proportional hazard models, the only independent factor predicting the risk of variceal rebleeding was treatment with EP (hazard ratio = 0.122, 95% CI 0.055–0.270, *p* = 0.000) (Table 2).

**Table 1:** Baseline characteristics of the patients

	EP group (*n* = 51)	E group (*n* = 51)	*p* value
Age (years)	52.26 ± 9.93	54.69 ± 9.73	0.22^a^
Gender, *n* (%)			
Male	27 (53%)	36 (71%)	0.10^c^
Female	24 (47%)	15 (29%)	
Etiology of cirrhosis, *n* (%)			
Hepatitis B virus	28 (55%)	27 (54%)	
Hepatitis C virus	3 (6%)	4 (7%)	
Alcoholic liver disease	3 (6%)	5 (12%)	0.68^c^
Autoimmune hepatitis	1 (2%)	5 (9%)	
Unexplained cause	16 (31%)	10 (18%)	
Child–Pugh score, *n* (%)	6.63 ± 1.20	6.77 ± 1.70	0.83^b^
Class A	29 (57%)	29 (57%)	
Class B	21 (41%)	19 (37%)	0.92^b^
Class C	1 (2%)	3 (6%)	
Degree of esophageal varices, *n* (%)			
F1	1 (2%)	1 (2%)	
F2	11 (22%)	5 (10%)	0.13^b^
F3	39 (76%)	45 (88%)	
White blood cell (× 10^9^/L)	2.73 ± 1.29	2.77 ± 1.47	0.89^a^
Red blood cell (× 10^12^/L)	3.21 ± 0.74	3.28 ± 0.81	0.66^a^
Platelet (× 10^9^/L)	61.08 ± 19.53	66.45 ± 18.26	0.15^a^
Hemoglobin (g/L)	86.18 ± 26.36	89.90 ± 26.02	0.47^a^
Prothrombin time (s)	15.11 ± 2.41	14.76 ± 1.95	0.43^a^
INR	1.33 ± 0.21	1.29 ± 0.17	0.31^a^
Prothrombin activity (%)	65.08 ± 13.22	66.31 ± 13.55	0.64^a^
ALT(U/L)	24.57 ± 18.71	40.80 ± 103.06	0.22^a^
AST(U/L)	29.71 ± 16.62	51.94 ± 124.14	0.21^a^
Alkaline phosphatase (U/L)	80.14 ± 35.21	96.88 ± 58.94	0.08^a^
Albumin (g/L)	36.43 ± 5.91	34.92 ± 6.55	0.22^a^
Total bilirubin (umol/L)	21.04 ± 15.85	20.68 ± 10.68	0.89^a^
Follow-up duration (months)	21.53 ± 3.14	20.87 ± 2.15	0.24^a^

Data are means ± standard deviations

*INR* international normalized ratio. *ALT* alanine aminotransferase, *AST:*aspartate aminotransferase.

^a^Determined with the *t* test

^b^Determined with Mann–Whitney *U* test

^C^Determined with the Chi-squared test

**Table 2:** Factors predicting variceal rebleeding

Variables	COX univariate analysis (*p* value)	COX multivariate analysis (*p* value)	Hazard ratio(95% CI)
Age	0.710	–	–
Gender	0.107	–	–
Etiology	0.773	0.795	–
Child–Pugh score	0.261	0.695	–
White blood cell	0.664	–	–
Red blood cell	0.400	–	–
Platelet	0.670	0.197	–
Prothrombin time	0.849	0.062	–
INR	0.699	0.076	–
Prothrombin activity	0.938	–	–
ALT	0.001	0.822	–
AST	0.002	0.802	–
Albumin	0.836	0.880	–
Total bilirubin	0.341	–	–
Treatment	0.009	0.000	0.122 (0.055–0.270)

*CI* confidence interval, *INR* international normalized ratio, *ALT* alanine aminotrans-ferase, *AST*: aspartate aminotransferase

### Variceal recurrence

During the follow-up, 11 patients (22%) in the EP group and 34 patients (67%) in the E group generated new varices that required further endoscopic prophylactic therapy. As demonstrated by the Kaplan–Meier analysis (Fig. 2b), the recurrence rate in the EP group was significantly lower than that in the E group (log-rank* p* < 0.001). Only one patient in the EP group and two in the E group generated new varices with form F2. The median variceal recurrence period was 11 months in the E group. The COX proportional hazard models revealed that treatment with EP (hazard ratio = 0.160, 95% CI 0.077–0.332, *p* = 0.000) was the only independent factor predicting the risk of variceal recurrence (Table 3).

**Table 3:** Factors predicting variceal recurrence

Variables	COX univariate analysis (*p* value)	COX multivariate analysis (*p* value)	Hazard ratio (95% CI)
Age	0.747		–
Gender	0.145		–
Etiology	0.988		–
Child–Pugh score	0.218	0.235	–
White blood cell	0.934		–
Red blood cell	0.323		–
Platelet	0.422	0.092	–
Prothrombin time	0.932	0.603	–
INR	0.706	0.213	–
Prothrombin activity	0.950	0.967	–
ALT	0.178		–
AST	0.202		–
Albumin	0.746	0.694	–
Total bilirubin	0.326		–
Treatment	0.003	0.000	0.160 (0.077–0.332)

*CI* confidence interval, *INR* international normalized ratio, *ALT* alanine aminotrans-ferase;,*AST *aspartate aminotransferase

### Chronological changes in the peripheral blood cell count

**Fig.3:**
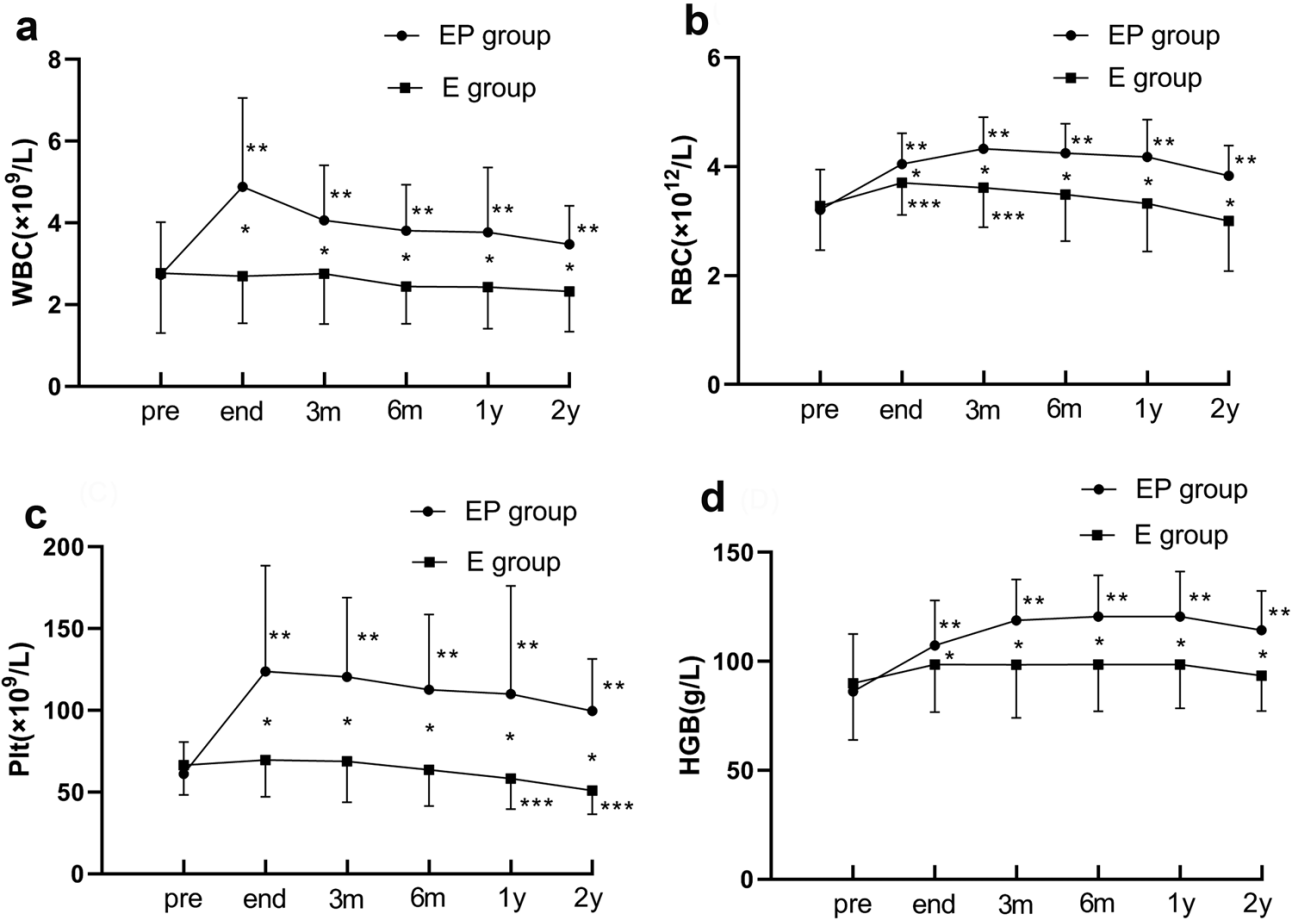
Follow-up results of peripheral blood cell count. **a** White blood cell (WBC), **b** red blood cell (RBC), **c** platelet (PLT), **d** hemoglobin (HGB) in EP group and E group. *PSE* partial splenic embolization. *There was a significant difference between the EP group and E group (*p* < 0.05). **It was significantly increased at this point than the pre-EP treatment (*p* < 0.05). ***It was significantly increased/decreased at this point than the pre-E therapy(*p* < 0.05)

The white blood cell (WBC) count, red blood cell (RBC) count, platelet (PLT) count, and hemoglobin (HGB) level in the EP group were significantly increased compared to those in the E group at any time point during the 2-year follow-up (*p* < 0.05; Fig. 3a–d).

The subgroup analysis revealed significant improvements in the peripheral blood cell count in the EP group during the follow-up. First, the WBC count increased significantly after the treatment and this increase was maintained for 2 years (*p* < 0.05; Fig. 3a). The WBC count increased from 27 to 79% during the follow-up compared to the baseline value. Simultaneously, the median RBC and the hemoglobin (HGB) level displayed a significant increment from 20 to 35% and from 25 to 40%, respectively, compared to the baseline value; these increments were maintained for 2 years (*p* < 0.05; Fig. 3b, d). In addition, the PLT count almost doubled from 61.08 ± 19.6 × 10^9^/L to a mean value of 123.76 ± 64.71 × 10^9^/L after the treatment. Although the PLT count decreased over time, it remained significantly elevated in comparison to the pre-procedural levels (*p* < 0.05; Fig. 3c). As for the E group, the WBC count, RBC count and HGB level showed no significant post-procedural difference at any time point. Moreover, the PLT count in the E group decreased significantly 1 year after the procedure (*p* < 0.05; Fig. 3c).

### Chronological changes in the liver function parameters

**Fig.4:**
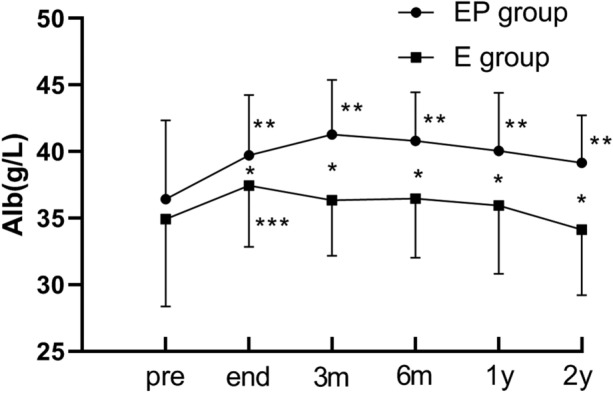
Follow-up results of albumin concentration. *PSE*, partial splenic embolization. *There was a significant difference between the EP group and E group(*p* < 0.05). **It was significantly increased at this point than the pre-EP treatment (*p* < 0.05). ***It was significantly increased at this point than the pre-E therapy (*p* < 0.05)

The albumin (ALB) concentration in the EP group increased significantly compared to that in the E group at any time point after treatment. In the E group, the ALB concentration decreased significantly 1 year after the procedure (*p* < 0.05; Fig. 4). The levels of alanine aminotransferase level (ALT), aspartate aminotransferase (AST), alkaline phosphatase (AKP), and total bilirubin exhibited no significant difference between the two groups at any time point.

### Chronological changes in the coagulation function

**Fig.5:**
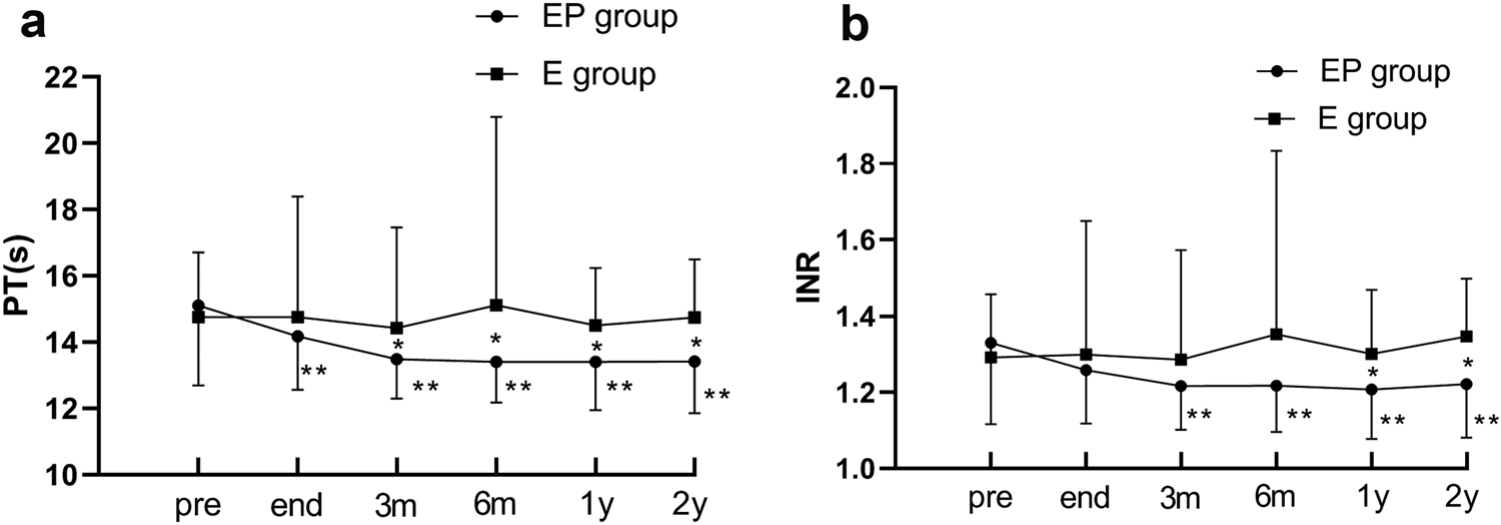
Follow-up results of coagulation function parameters. **a** prothrombin time (PT), **b** international normalized ratio (INR), **c** prothrombin activity (PTA) in EP group and E group. *PSE* partial splenic embolization. *There was a significant difference between the EP group and E group (*p* < 0.05). **It was significantly increased/decreased at this point than the pre-EP treatment (*p* < 0.05)

The coagulation function parameters, such as the prothrombin time (PT) (Fig. 5a), international normalized ratio (INR) (Fig. 5b), and prothrombin activity (PTA) (Fig. 5c), in the EP group showed significant improvement after the treatment when compared to those in the E group. Moreover, the EP treatment significantly improved the coagulation function parameters at any time point during the follow-up (*p* < 0.05; Fig. 5). The median INR value decreased significantly over time from 1.33 before PSE to 1.20 over 2 years. The median PT also showed a significant decrease from 15.11 s before PSE to 13.40 s over 2 years. The value of PTA in the EP group gradually increased by 22% compared to the baseline.

### Child–Pugh class/score

The Child–Pugh class/score in the EP group exhibited significant improvement compared to the baseline and this improvement persisted for 2 years. Indeed, the EP group presented a significantly reduced Child–Pugh score 6 months after the treatment compared to the E group (Table 4).

**Table 4:** Follow-up results of Child–Pugh class/score

Time	Child–Pugh class		Child–Pugh score			
	EP group(A/B/C)	E group(A/B/C)	*p*	EP group	E group	*p*
Pre-treatment	29/21/1	29/19/3	0.866	6.00(1)	6.00(3)	0.702
End of the treatment	39/12/0*	34/16/1	0.254	6.00(1)*	6.00(2)*	0.904
Post of the treatment						
3 months	43/7/0*	38/11/2	0.132	5.00(1)*	6.00(2)*	0.131
6 months	45/4/0*	36/15/0	0.007	5.00(1)*	6.00(2)	0.074
1 year	41/8/0*	27/16/2	0.009	6.00(1)*	6.00(2)	0.008
2 years	27/7/0*	15/13/3	0.006	6.00(1)*	7.00(2)	0.025

Data are median (Interquartile Range)/M (IQR) or numbers of patients. Statistical analysis was performed using Mann–Whitney *U* test

*It was significantly different at this time point than pre-treatment

### Changes in the portal vein diameter and times of endoscopic treatment

The diameters of the main portal vein (MPV) measured using CT angiography did not exhibit any significant difference (*p* > 0.05) before PSE (20.41 ± 4.14 mm) and after PSE (18.81 ± 3.44 mm). The times of EVL in the EP group (1.98 ± 0.81) were significantly reduced compared to those in the E group (3.65 ± 0.10). Moreover, the bands assumption in the EP group (14.57 ± 7.47) were significantly lower than those in the E group (25.59 ± 8.49). The times of cyanoacrylate injection in the EP group (1.31 ± 0.62) were significantly reduced than those in the E group (1.82 ± 1.01). Furthermore, the glue assumption in the EP group (2.06 ± 2.15) was significantly reduced than that in the E group (3.93 ± 4.02).

### Adverse events

After the endoscopic treatment, about one-third of the patients experienced endoscopy-related adverse events such as retrosternal pain (28%), nausea (12%), moderate fever (below 39.0 °C; 4%), which usually palliated spontaneously after 2–3 days without any special management. Two patients in the E group experienced post-band ligation hemorrhage as detected in endoscopy. The hemorrhage was controlled pharmacologically after hospitalization. After the PSE procedure, the majority of the patients exhibited at least one symptom of the post-embolic syndrome, such as abdominal pain (74%), abdominal distention (56%), nausea (7%), and fever (below 39.0 °C; 65%); the symptoms were managed medically prior to discharge. One patient with Child–Pugh Class B developed portal vein thrombosis that was observed during a CT angiography examination 3 weeks after PSE. This patient was treated with anticoagulant for 6 months, which completely recanalized the portal vein. No severe complications, such as splenic abscess, splenic rupture, serious infection, and sepsis, were observed post-PSE. Mortality related to the endoscopic therapy or the PSE procedure was not recorded during the follow-up.

## Discussion

Liver cirrhosis patients, especially those with additional complications such as severe hypersplenism and thrombocytopenia and those who survive acute variceal bleeding, exhibit a high rate of rebleeding and mortality. However, the optimal management of variceal hemorrhage in patients with hypersplenism is not well understood [5]. Therefore, we first attempted using the EP treatment for the management of variceal rebleeding in a multicenter randomized controlled trial. Our results showed that the EP treatment could significantly reduce the variceal rebleeding and recurrence rate compared to the E treatment. Moreover, the EP treatment resulted in a significant long-term improvement in the peripheral blood cell count, liver function and coagulation function.

Treatments such as endoscopy, portosystemic shunts, and pharmacotherapy are attempted to decrease the risk of rebleeding and mortality [13]. Currently, the first-line therapy for the secondary prophylaxis of esophageal variceal hemorrhage is EVL plus NSBB [3]. However, adopting only endoscopic therapy as a treatment to eradicate varices cannot fundamentally relieve portal hypertension nor can it reduce the risk of rebleeding. Varices often recur with a 50% rate at 2 years, since the portal pressure and blood flow remain unchanged [14]. Several researchers have demonstrated that the portal pressure is positively correlated with the spleen size and that portal pressure can induce splenomegaly. Splenomegaly and hypersplenism are common in patients with cirrhosis, both usually accompanying each other [15, 16]. In cirrhosis patients with hypersplenism, the extremely low platelet count may promote rebleeding [17]. Regardless of these factors, the primary goal of variceal management should be the prevention of variceal rebleeding and recurrence after variceal eradication.

In a series of clinical trials, PSE alone or in combination with conventional therapies was reported to be beneficial in reducing the risk of variceal bleeding and variceal recurrence in cirrhosis patients [18–20]. However, no research has been conducted on the secondary prophylaxis management of gastroesophageal variceal hemorrhage in cirrhosis patients with hypersplenism. Our study showed that the EP treatment was significantly associated with a lower incidence of variceal bleeding and variceal recurrence. Moreover, group assignment emerged as the only predictor for variceal bleeding and variceal recurrence. It was observed that after the EP treatment, the diameter of MPV had decreased, although the difference was not significant (*p* > 0.05). The procedure times of EVL, cyanoacrylate injection, and the assumption of bands and glue were significantly reduced in the EP group (*p* < 0.05). The reduction in time could be attributed to portal decompression after PSE, which unfortunately cannot be confirmed as we did not measure the portal vein pressure in this study. In the presence of collateral circulation, especially esophageal varices, both splenic and superior mesenteric arteries exhibit significantly high flow volumes. Around 60–80% dearterialization occurs after PSE, and the splenic venous outflow and pressure decreases, following which the left and short gastric veins of the varices collapse [21]. Hence, after PSE, the frequency of endoscopic procedures decreases and management by endoscopic physicians become easier. PSE was reported to reduce the size of the spleen, splenic blood flow, splenic venous pressure, and portal venous pressure, and improve the hepatic arterial hypertensive state in cirrhotic patients, similar to the findings of the present study [22]. Furthermore, the improvement in portal hemodynamics is often associated with reduced bleeding from the esophageal or gastric varices. Our results showed that the EP group reduced the variceal rebleeding rate by 48.4% and the variceal recurrence rate by 67.2% compared to the rates observed in the E group. Thus, the EP treatment is indeed better for the management of the secondary prophylaxis of VH and the reduction of variceal bleeding.

The current study indicated that both PLT and WBC counts were significantly increased within 2 weeks of PSE. Moreover, the cirrhosis patients with more than 50% splenic infarction presented a long-term improvement in leukocytopenia and thrombocytopenia, although the peripheral blood cell count declined consistently over time [23–25]. In our study, we demonstrated again that the WBC and PLT counts increased significantly within weeks of undergoing PSE. However, the counts decreased consistently in the subsequent months and years, although they were significantly better than those at the baseline. Moreover, the RBC count and the HGB level were also significantly elevated in the two groups, especially after the EP treatment.

The immediate improvement in the WBC count due to PSE could be attributed to the release of white cells from the spleen, considering it as an acute inflammatory response. The long-term improvement in the WBC count in the EP group could be ascribed to an improvement in the blood-producing function of the bone marrow after PSE [26, 27]. The PLT count might have increased due to improvement in the hepatic production of thrombopoietin (TPO) after PSE, as well as due to the reduction in the splenic platelet pool and increase in the platelet survival time [28]. Interestingly, our study found that both the evaluated treatments, majorly the EP treatment, significantly improved the hemoglobin level and the RBC count for a long time. The hemoglobin level and the RBC count are relatively difficult to interpret, since they are influenced by transfusions, the confounding variance of variceal bleeding, and the effect of erythrocyte survival time. It is reported that RBC destruction occurs in the enlarged spleen at a rate of 0.5%–4.4% of the total RBC mass/day, with no evidence of immune hemolysis [29]. Increased hemoglobin level and RBC count in our study were attributed to the synergistic effects of PSE and endoscopic therapies. An increase in the erythrocyte survival time, decrease in the destruction of RBCs by monocytes and macrophages, activation of the bone marrow hematopoiesis after PSE, and decrease in the bleeding episodes after the EP treatment could all have led to the elevated hemoglobin levels and RBC count [26, 30].

Furthermore, our study demonstrated that the EP treatment is a potential therapeutic tool for improving therapeutic tool to improve liver function and coagulation function. The serum ALB concentration in the EP group increased by 9–13% compared to the baseline and was maintained at > 39 g/L for 2 years. We found no significant changes in the other liver function parameters such as ALT, AST, AKP, and total bilirubin, which are more reflective of hepatocyte damage. As for the coagulation function indices, the EP group presented a remarkable decrease in PT and INR and a significant increase in PTA. The parameters that improved in the EP group included albumin, PT, and PTA, which are all indicators of the liver’s ability to synthesize proteins. In cirrhosis patients, especially those in the decompensated stage, the hemostatic function is often disrupted, because most pro- and anticoagulant proteins are synthesized in the hepatic parenchymal cells. The improvements in PT, PTA, and INR in the EP group demonstrated that this treatment could not only improve the hepatic protein synthetic capabilities but could also maintain the coagulation and anticoagulation homeostasis.

The precise mechanisms of the EP treatment underlying the improvement in the liver function and coagulation function are currently unclear, although it is reported that they may be related to the hemodynamic alterations and changes in the host immunological factors [30–33]. We observed a decreased splenic venous flow and an increased flow of both hepatic artery and superior mesenteric artery under DSA after PSE. The decrease in the portal blood flow and a relative increase in the gastrointestinal-derived blood supply post-PSE may increase the supply of nutrients and cytokines to the liver. Research shows that an immunological mechanism may also be involved in the improvement in the liver function. An increased amount of PCNA-positive hepatocytes was observed in the post-PSE liver. These cells are reported to exhibit high proliferative activity, indicating that PSE may induce a liver regenerative response [27]. In addition, PSE may induce the activation of host immunity via complicated interactions between T cells and cytokines, and thereby improving liver function and coagulation function.

As for all research, the present study also had certain limitations. First, the follow-up period was short. Thus, a study with a long-term follow-up is needed to verify our findings and assess the long-term survival prognosis. Second, we did not measure the portal pressure and hemodynamics after the PSE. Nonetheless, based on our multiple positive results, the application of PSE combined with conventional endoscopic therapies could be included in the clinical guidelines for the secondary prophylaxis management of VH. Third, the safety and efficiency of the EP treatment in cirrhotic patients with Child–Pugh score C was not fully assessed. Future studies with Child–Pugh score C patients are needed to verify this aspect.

In conclusion, the EP treatment is effective in reducing variceal bleeding and variceal recurrence. Moreover, it can chronically increase the peripheral blood cell count and improve the liver function, coagulation function, and the Child–Pugh score. Therefore, EP treatment proposed as an effective therapy superior to the conventional endoscopic therapies for the secondary prophylaxis of VH in cirrhosis patients with hypersplenism.

## Supplementary Information

Below is the link to the electronic supplementary material.

Supplementary file1 (DOCX 44 KB)

## Data Availability

All date generated or analyzed during this study are included in this published article.

## Abbreviations

EV: Esophageal varices
EVL: Endoscopic variceal ligation
GEVs: Gastroesophageal varices
GV: Gastric varices
NSBB: Non-selective beta-blocker
PSE: Partial splenic embolization
VH: Variceal hemorrhage
