# Supplementary material for: Partial splenic embolization combined with endoscopic therapies and NSBB decreases the variceal rebleeding rate in cirrhosis patients with hypersplenism: a multicenter randomized controlled trial
Source: Hepatol Int. 2021 Feb 27;15(3):741–52. doi: 10.1007/s12072-021-10155-0 (PMC8286949; doi:10.1007/s12072-021-10155-0)
Supplement: Supplementary file 1 — Supplementary file1 (DOCX 44 KB) [file 12072_2021_10155_MOESM1_ESM.docx]

**STUDY PROTOCOL**

**Authors Statement:**

This study protocol was subject to critical review and has been approved by all the authors of this study.

**Title of study:**

Partial splenic embolization combined with endoscopic therapies and NSBB decreases the variceal rebleeding rate in cirrhosis patients with hypersplenism: a multicenter randomized controlled trial.

**Background:**

Varices and variceal hemorrhage (VH) are major complications of portal hypertension that cause high mortality in cirrhosis patents. Nearly half of the newly diagnosed patients with compensated cirrhosis have developed GEVs. Furthermore, the incidence is higher in patients with severely impaired liver function (Child-Pugh class B/C).[[1](#_ENREF_1)] The risk of first variceal hemorrhage is approximately 5-15% yearly, which increases with impaired liver function, especially the size of varices and presence of red colour signs over the varices.[[2](#_ENREF_2)] In spite of the recent progress, variceal bleeding as a medical emergency is still associated with a 6-week mortality of 10–20% .[[3](#_ENREF_3)] The 1-year risk of rebleeding after the first episode of bleeding is 60% and the mortality rate is up to 33%.[[4](#_ENREF_4),[5](#_ENREF_5)] Hence, the secondary prophylaxis is necessary to prevent rebleeding for patients who have recovered from an episode of acute VH.

Currently, the research and resulting guidelines about secondary prophylaxis of VH in cirrhosis patients during these years have led to improvements in the care and survival of patients. According to the BavenoⅥrecommendations, non-selective beta-blockers (NSBB) combined endoscopic variceal ligation (EVL) is recommended for the secondary prophylaxis of esophageal variceal (EV) hemorrhage . Cyanoacrylate injection in patients with large GEVs type 2 or isolated gastric varices type 1 is promising for the prevention of the first variceal bleeding.[[3](#_ENREF_3)] A retrospective cohort study demonstrated the efficacy of continued EVL plus cyanoacrylate injection for secondary prophylaxis of variceal bleeding in patients with concomitant EV and gastric varices (GV).[[6](#_ENREF_6)] But applying cyanoacrylate in those patients need further studies to evaluate the risk/benefit ratio.

The incidence of hypersplenism in patients with cirrhosis and portal hypertension has been reported to range from 11 -55%.[[7](#_ENREF_7)] Hypersplenism often develops in parallel with splenomegaly. Clinically, splenomegaly has been associated with not only the maintenance of portal hypertension by the increase in splenic blood flow but also a poor prognosis in patients with liver cirrhosis.[[7](#_ENREF_7)] Hypersplenism is a major contributing factor in the development of thrombocytopenia, anemia, and leukopenia in cirrhotic patients. As a result of the increased risk of spontaneous bleeding, thrombocytopenia limits numerous clinical strategies, such as surgical and antiviral therapy. In addition, thrombocytopenia and splenomegaly are independent predictors of bleeding from large varices.[[8](#_ENREF_8)] PSE is efficacious in improving hematologic parameters, reducing episodes of variceal bleeding, maintaining the long-term eradication of varices, and enhancing hepatic synthesis.[[9](#_ENREF_9),[10](#_ENREF_10)] Unfortunately, no universally accepted therapy has been established for GVEs in patients with hypersplenism, especially those with thrombocytopenia. Also, the research of endoscopic therapies+ PSE treatment for GEVH is still rare worldwide.

Therefore, we prospectively conducted a study to assess the efficacy of EVL and cyanoacrylate injection combined PSE versus EVL and cyanoacrylate injection alone for secondary prophylaxis of VH in cirrhosis patients with severe hypersplenism.

**Objectives:**

Evaluate and compare the efficacy and safety of endoscopic treatment plus PSE (EP) treatment versus endoscopic (E) treatment in the secondary prophylaxis of VH for cirrhosis patients with hypersplenism.

**Primary endpoint:**

The primary endpoint of the study was variceal rebleeding during 2 years follow-up.

**Second endpoints:**

The second endpoints were severe variceal recurrence and mortality. Hematological index, serum biochemical parameters and Child-Pugh scoring system were measured at each time point.

**Study centers:**

Qi Lu Hospital of Shandong University, the 960th Hospital of the PLA Joint Logistice Support Force, Shandong Provincial Hospital.

**Studied period (years):**

Estimated date first subject enrolled: June 2016

Estimated date last subject enrolled: June 2019

Estimated study completion date: December 2019 (including all follow-up assessments)

**Methodology:**

Prospective, multicenter, double-blind, randomize controlled trial.

Patients were randomly assigned to two groups according to a computer-generated randomization sequence with 1:1 ratio.

**Endoscopic group (E group):**

Participants underwent continued endoscopic variceal ligation (EVL) for esophageal varices and cyanoacrylate injection for gastric varices (collectively referred to endoscopic treatment) until complete endoscopic eradication of the varices.

**Endoscopic treatment plus PSE group (EP group):**

Participants underwent PSE 1 week before after endoscopic treatment and received subsequently continued endoscopic treatment until complete endoscopic eradication of the varices.

Standard dose of NSBB ([propranolol](javascript:;) ) is applied in patients according to recommendations in BavenoⅥ if there are no contraindications. The dosage of [propranolol](javascript:;)was titrated to reduce resting heart rate by 25% or the heart rate up to 55beats/min., or up to the maximum tolerated dose. All post-hepatitis B cirrhotic participants received anti-viral therapy.

**Main inclusion criteria:**

The enrollment criteria were: (i) patients aged between 18 and 75 years; (ii) patients who had recovered from an episode of VH or patients who had survived from acute VH and the bleeding had stopped for five days; (iii) patients with a diagnosis of liver cirrhosis and portal hypertension on clinical examination, laboratory test, and imaging or histological examination; and (iv) patients with hypersplenism and thrombocytopenia (platelets <100,000/µL).

**Main exclusion criteria:**

The exclusion criteria were: (i) previous therapy (splenectomy, PSE, EVL, tissue adhensive injection , or usage of NSBB ) to prevent rebleeding; (ii) bleeding from isolated gastric or ectopic varices; (iii) hepatocellular carcinoma or other malignant tumors; (iv) contraindications for the use of NSBBs, hepatic failure or Child-Pugh class C; (v) hepatic failure; (vi) contraindications for PSE; (vii) pregnancy and lactation; and (viii) inability to sign informed consent.

**Clinical study flow:**

1. Selection and enrollment of the patients
2. Selecting suitable patients according to the inclusion and exclusion criteria.
3. Informing the aims of this study and patients signing informed consent before their participation.
4. Collecting demographic data of the patients, including age, sex, height, weight, etc.
5. Collecting medical history of the patients, including history of present illness, previous medical history, surgical history, etc.
6. Completing physical examination of the patients.
7. Completing laboratory tests: [blood](javascript:;) [routine](javascript:;) [examination](javascript:;); [routine](javascript:;) [urine](javascript:;) [test](javascript:;) ; stool routine [examination](javascript:;); liver function test; renal function test; coagulation function test; infectious disease test series.
8. Completing ECG; completing abdominal enhanced CT scan or abdominal enhanced MRI.
9. Treatment for patients
10. E group: patients underwent continued endoscopic variceal ligation (EVL) for esophageal varices and cyanoacrylate injection for gastric varices (collectively referred to endoscopic treatment). After the initial session, EVL was performed at regular intervals about 4 weeks and if necessary, additional cyanoacrylate was injected into distinct large GV until the varices were eradicated. The duration of treatment is approximately 12-16weeks.
11. EP group: patients underwent PSE 1 week before endoscopic treatment and received subsequently continued endoscopic treatment until complete endoscopic eradication of the varices. The duration of treatment is approximately 13-17weeks.
12. Follow-up:

After variceal eradication was achieved, the routine follow-up was performed in each patient at 3 months, 6 months, 1 year and 2 years. The follow-up visit included clinical assessment, endoscopic follow-up, hematological parameters, serum biochemical analysis and Child-Pugh scoring system. Follow-up endoscopy was performed every 3 months and then moved to every 6 months if there was no recurrence. Once the patients suffered from variceal bleeding or generated new varices with F2/F3 approved by endoscopy, then the patients would be dropped out from the group.

1. The clinical assessment data were collected through the follow up in the outpatient department from the patients. Clinical signs of upper gastrointestinal hemorrhage, such as hematemesis, coffee ground vomitus, hematochezia, or melena; general condition; infection status; complications after PSE, such as splenic abscess, abdominal infection, pneumonia, prolonged fever were carefully recorded.
2. Follow-up endoscopy was performed every 3 months and then moved to every 6 months if there was no recurrence. If rebleeding occurred the emergency endoscopy was performed to identify the bleeding source. If GVEs bleeding were responsible, then the endoscopic therapies were performed again.
3. Hematological and serum biochemical analysis (Table 1) including white blood cell count, red blood cell count, platelet count, hemoglobin level, albumin level, aspartate aminotransferase level, alanine aminotransferase level, alkaline phosphatase level, total bilirubin level, prothrombin time, international normalized ratio, prothrombin activity level, and those index were repeated in the follow-up periods.

Table1. Hematological and serum biochemical index

| white blood cell | alanine aminotransferase |
| --- | --- |
| red blood cell | total bilirubin level |
| platelet | prothrombin time |
| hemoglobin | international normalized ratio |
| albumin | prothrombin activity |
| aspartate aminotransferase |  |

1. Child-Pugh scoring system includes Child-Pugh score and Child-Pugh degree. The Child-Pugh scoring system will estimated at the time point during follow-up.

**Study end point:**

The end points of this study were variceal rebleeding, death, severe variceal recurrence, two years after treatment, and time to the completion date.

**Statistical methods:**

1. Sample size: (planned)

The total sample size is calculated according to the [statistical](javascript:;) [method](javascript:;) of multi-center two group clinical trials. The number of participants included in each hospital is determinate by local medical level.

Estimation formula

$$n_{1}=n_{2}=\frac{[Z_{\alpha}\sqrt{2p\left( 1-p \right)}+Z_{\beta}\sqrt{p_{1}\left( 1-p_{1} \right)+p_{2}(1-p_{2})}]}{{(p_{1}-p_{2})}^{2}}$$

According to previous studies, the rebleeding rate in the E group was approximately p_1_=0.37, and the rebleeding rate in the EP group was approximately p_2_= 0.15. We set α=0.05，β=0.10，u_0.05/2_=1.96, u_0.10_=1.282. So , n_1_=n_2_= 46. Ultimately , the sample size is approximately 110 patients when considering considering a 20 % dropout rate. Qi Lu Hospital of Shandong University will enroll 2/3 of the patients (approximately 74 patients), the 960th Hospital of the PLA Joint Logistice Support Force and Shandong Provincial Hospital will enroll 1/3 of the patients (approximately 36 patients).

1. Evaluation of the primary objective

The varices recurrence rate or varices rebleeding rate will analyzed by Kaplan–Meier survival curve and the log-rank test will used to compare the difference between the groups. The Cox proportional hazard analysis models will used to assess the potential risk factors of varices recurrence or varices rebleeding. The patients will provide 90% power to detect a statistically significant (*p* <0.05 two-sided test) difference between the two treatment groups.

1. Evaluation of the secondary objective

The variables of hematological and serum biochemical index will be analyzed using [variance](javascript:;) [analysis](javascript:;). Then the two-sample Student t tests or Mann-Whitney U test will used to compare the difference between the groups. On subgroup analysis, the change from baseline in variables of hematological and serum biochemical index will be analyzed using the same method as the two groups. The patients will provide 90% power to detect a statistically significant (p 0.05 two-sided test) difference between the two treatment groups.

**Adverse reaction:**

The patients would suffered from nausea, vomiting, abdominal distension, abdominal discomfort, gastrointestinal bleeding, perforation or other adverse reactions when underwent endoscopic treatment. The patients would suffer from abdominal pain, fever, nausea, vomiting, etc. We will treat patients symptomatically when they suffered from mild adverse reactions. If patients suffered from serious adverse reactions, all therapeutic measures will be terminated.

**Ethics:**

This prospective multicenter randomized controlled study was conducted conforming to the Ethical Guidelines of the 1975 Declaration of Helsinki (6th revision, 2008) and approved by the Ethics Committee of Shandong University. This study was register on the website ClincialTrials.gov, number, NCT02778425. All the information of patients was anonymized prior to the analysis.

**Reference:**

1. Garcia-Tsao G, Sanyal AJ, Grace ND, Carey W, Practice Guidelines Committee of the American Association for the Study of Liver D, et al. (2007) Prevention and management of gastroesophageal varices and variceal hemorrhage in cirrhosis. Hepatology 46: 922-938.

2. Brunner F, Berzigotti A, Bosch J (2017) Prevention and treatment of variceal haemorrhage in 2017. Liver International 37: 104-115.

3. de Franchis R, Baveno VIF (2015) Expanding consensus in portal hypertension: Report of the Baveno VI Consensus Workshop: Stratifying risk and individualizing care for portal hypertension. J Hepatol 63: 743-752.

4. Puente A, Hernandez-Gea V, Graupera I, Roque M, Colomo A, et al. (2014) Drugs plus ligation to prevent rebleeding in cirrhosis: an updated systematic review. Liver Int 34: 823-833.

5. Bosch J, García-Pagán JC (2003) Prevention of variceal rebleeding. The Lancet 361: 952-954.

6. Chen J, Zeng XQ, Ma LL, Huang XQ, Tseng YJ, et al. (2016) Long-term efficacy of endoscopic ligation plus cyanoacrylate injection with or without sclerotherapy for variceal bleeding. J Dig Dis 17: 252-259.

7. Li L, Duan M, Chen W, Jiang A, Li X, et al. (2017) The spleen in liver cirrhosis: revisiting an old enemy with novel targets. J Transl Med 15: 111.

8. Giannini E, Botta F, Borro P, Risso D, Romagnoli P, et al. (2003) Platelet count/spleen diameter ratio: proposal and validation of a non-invasive parameter to predict the presence of oesophageal varices in patients with liver cirrhosis. Gut 52: 1200-1205.

9. N'Kontchou G, Seror O, Bourcier V, Mohand D, Ajavon Y, et al. (2005) Partial splenic embolization in patients with cirrhosis: efficacy, tolerance and long-term outcome in 32 patients. Eur J Gastroenterol Hepatol 17: 179-184.

10. Sangro B, Bilbao I, Herrero I, Corella C, Longo J, et al. (1993) Partial splenic embolization for the treatment of hypersplenism in cirrhosis. Hepatology 18: 309-314.
